# Origin of the largest South American transcontinental water divide

**DOI:** 10.1038/s41598-018-35554-6

**Published:** 2018-11-21

**Authors:** Alexandre Cunha Ribeiro, Claudio Riccomini, Jayme Alfredo Dexheimer Leite

**Affiliations:** 1 Departamento de Biologia e Zoologia, Instituto de Biociências, Universidade Federal de Mato Grosso, Av. Fernando Corrêa da Costa, 2367 - Boa Esperança, Cuiabá, MT 78060-900 Brazil; 2 Instituto de Geociências (IG) & Instituto de Energia e Ambiente (IEE), Rua do Lago, 562, Cidade Universitaria, 05508-080 - Universidade de São Paulo, Brazil, São Paulo, SP Brazil; 3 Departamento de Recursos Minerais, Faculdade de Geociências, Universidade Federal de Mato Grosso, Av. Fernando Corrêa da Costa, 2367 - Boa Esperança, Cuiabá, MT 78060-900 Brazil

## Abstract

Interbasin arches between hydrographic systems have a heterogeneous geological origin, forming under the influence of several different geomorphological processes. Independent of the underlying processes, these arches compartmentalize present-day river basins, encompassing different water chemistries, habitat types, soil domains, potential energy and, on a geological/evolutionary time scale, aquatic life varieties in the ecosystem. Through most of its length, the water divide between the Amazonian, Paraná-Paraguay, and São Francisco river basins in central South America coincides with an Upper Cretaceous intracontinental igneous alkaline province. This magmatism, independent of its nature, caused intense crustal uplift and influenced hydrological networks at different scales: from continental-scale crustal doming to continental break-up, and finally to local-scale phenomena. The available ages for alkaline rocks indicate a well-defined time-interval between 72.4 to 91 Ma (concentrated between 76 and 88 Ma) period of uplift that contributed to large-scale drainage compartmentalization in the region. Here we show that uplift associated with intrusive magmatism explains the origin and maintenance of the divide between the Amazonian, Paraná-Paraguay, and São Francisco river basins.

## Introduction

The age of a river system is a concept that is elusive in fluvial geology^1^. Theories regarding the age and existence of river systems include the following. (1) A river is at least as old as the onshore or offshore deposits that are related to it, such as delta or submarine fan deposits or a major erosional feature such as a submarine canyon. (2) A river is at least as old as the last major marine regression from its watershed. (3) The origin of a river can be dated back to the last major tectonic, glacial, and volcanic events affecting its drainage system^1^.

River basins are complex natural systems geographically limited by interbasin arches formed by different geological and geomorphological processes. Extremely close spatial correspondence between proposed mantle plume locations (associated with large igneous provinces and continental breakups) and several present-day drainage systems worldwide suggests a direct genetic relationship between magmatism and the origin of older drainage systems due to large crustal doming preceding continental break-up^2^. For example, the origin of several hydrographic systems in both Africa and South America can be dated back to the Early Cretaceous, coeval with the early opening of the South Atlantic Ocean^2^. The origin of present-day limits of major drainage basins could also be influenced by regional uplift in parts of South America during the late Cretaceous, as proposed by recent studies, which was related to the rapid spreading rates in South Atlantic^3^. Another line of research used South American river profiles (considered as a spatial and temporal function of regional uplift) to model the evolution of drainage during the last ~35 Ma as a result of the effects of dynamic topography^4^. These studies, however, do not rescue the original idea of the possible influence of intraplate magmatism on regional uplifts.

While the geomorphological impact of intracontinental magmatism has not been considered previously, paleodrainage reconstructions have been conducted for the convergent South American continental margin, showing well-supported geological evidence of Miocene marine incursions^5,6^, mega-wetlands^7^, and other fluvial systems associated with the Andean foreland^8–11^. Evidence of recent major hydrographic changes, such as the origin of the Pantanal wetland, Brazil, in the Cenozoic, have also been obtained^12–14^. However, geological processes related to the origin of inland river basins are not well known^15^, and this holds true for the water divide comprising the Amazonian, Paraná-Paraguay, and São Francisco river basins.

Geologically, the headwater streams of these continental-scale river systems are located at the margins of major South American cratons, namely the Amazonian, São Francisco, Rio de la Plata, São Luiz, and Luiz Alves cratons, which are surrounded by large ancient orogenic belts (Mantiqueira and Tocantins provinces) formed during the amalgamation of the Western Gondwana supercontinent in the Neoproterozoic^16^. Despite their orogenic origins, such ancestral mountains are too old to be directly associated with present-day landscapes or divides. However, a remarkable fact about Brazilian relief is the presence of Mesozoic summit surfaces at high altitudes^17^. Such flat tops on several high-relief relict topographic structures along the abovementioned water divide provide evidence of the long denudation history of an ancient Gondwanaland plateau^17^. The development of this Cretaceous mega-plateau of about 2000 m of topographic elevation^18^ was coeval with local-scale volcanism, rifting, and uplifts^19^. Installation of the present-day observed drainages occurred alongside such mega-geomorphological dynamism.

## Results

### Current Amazonian-Paraná-Paraguay-São Francisco water divide

**Figure 1:**
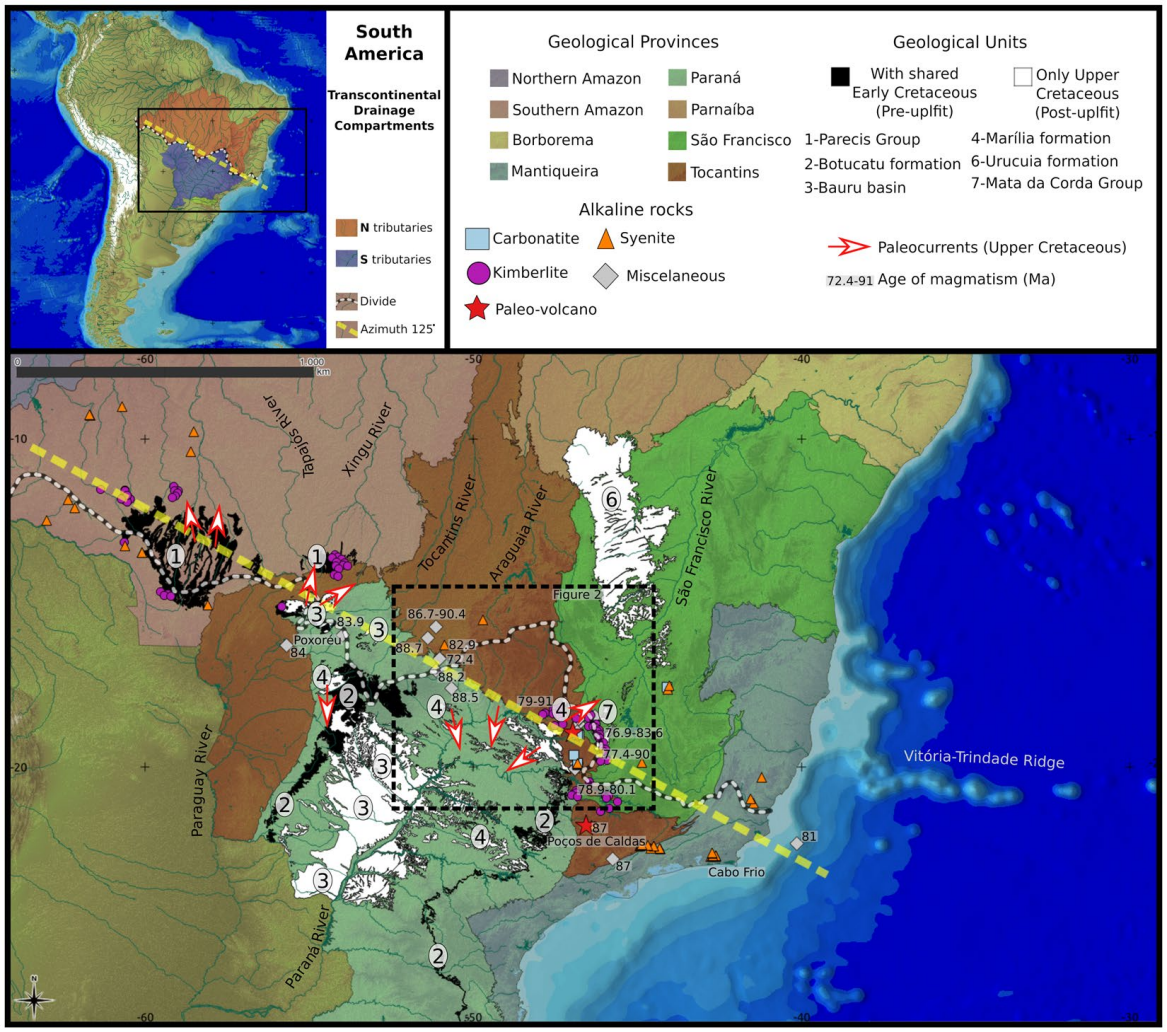
Major geological features associated with the Azimuth 125° lineament: topographic high-relief consisting of water divides between the Amazonian, São Francisco (draining northward) and Paraná-Paraguay systems (draining southward). Intrusive alkaline complexes, paleo volcanoes, paleocurrents, and ages of alkaline intrusions are also plotted. Geological units correspond to sedimentary rocks deposited before uplift, (occurring both northern and southern to Azimuth 125° lineament, in black), and those deposited after uplift (restricted to northern or southern sides, in white). Area illustrated in Fig. 2 limited by square. (image created with QGIS version 2.18.17, available in https://qgis.org/en/site/. Including data are: SRTM30 available in https://earthexplorer.usgs.gov/, GEOSGB data available in http://geosgb.cprm.gov.br/; GIS data provided by CPRM available in http://www.cprm.gov.br/publique/cgi/cgilua.exe/sys/start.htm?infoid=3489&sid=58, and http://geowebapp.cprm.gov.br/ViewerWEB/index_recmin.html).

**Figure 2:**
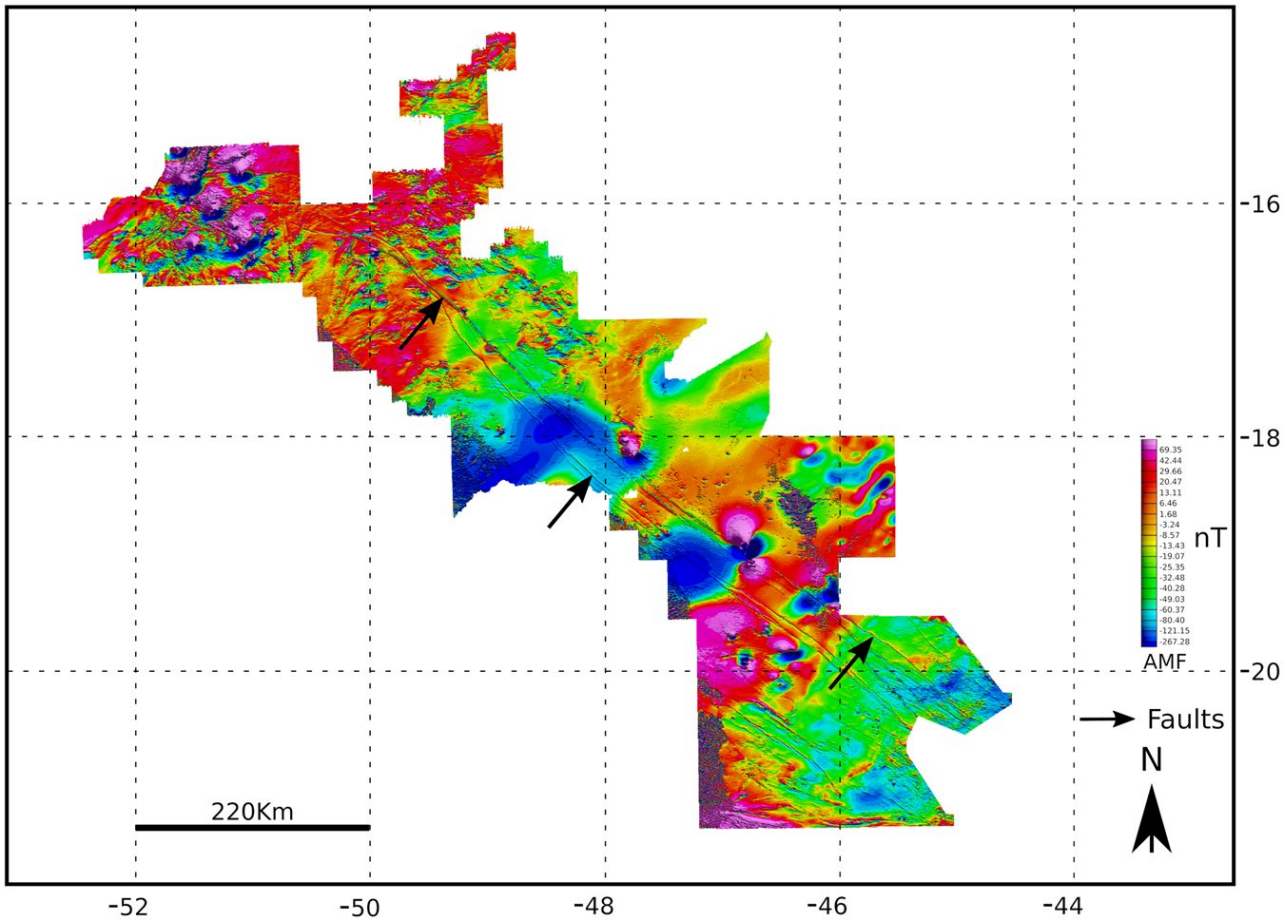
Azimuth 125° lineament based on earth’s anomalous magnetic field (AMF) modified from the literature^40^ (used with permission of Elsevier, license # 4376530981073).

One of the most conspicuous characteristics of the current configuration of the Amazonian, Paraná-Paraguay, and São Francisco river basins is the ~2300-km-long, NW-SE-oriented water divide. This long trajectory over the Brazilian shield coincides with a remarkable geological feature - the Azimuth 125° lineament (Figs 1 and 2). This feature was first described as a succession of diamond deposits, located in Brazil, aligned from Abaeté (state of Minas Gerais) to Rio Machado (state of Rondônia) within a NW-SE-oriented belt that is 1800 km long and approximately 200–300 km wide^20^. Another study proposed that Azimuth 125° extends from the state of Rondônia in the west to the state of Rio de Janeiro on the SE coast of Brazil^21^. Furthermore, this azimuth comprises one of the most significant set of faults that operated as a conduit for kimberlite, carbonatite, syenite, and several other alkaline magmas in Brazil (Fig. 2)^22^.

### Age of the central South American river basins

Previous contributions dealing with the origins of the modern river system in the South American interior^1,2,23,24^ agree with some basic points: (1) the present main water divides and basin architecture are Mesozoic in age; (2) major Jurassic-Cretaceous events, such as the break-up of the Gondwanaland, have a significant tectonic influence on the compartmentalization of present-day sedimentary and fluvial systems; and (3) the Andean chain significantly contributes to major hydrological changes; for example, Cenozoic deformations of the ancient post-Cretaceous paleo-plateaus were influenced by the geotectonic evolution of Andean foreland systems.

Sedimentary records of intracratonic basins (and associated paleocurrent data) provide insights on the timing of spliting between adjacent fluvial systems. Along the Azimuth 125° lineament, the youngest shared sedimentary sequence between northern (Parecis) and southern (Paraná) intracratonic sedimentary basins is the Lower Cretaceous sandstone of the Botucatu Formation, located at the western limit of the azimuth between the upper Tapajós-Xingú and Paraguay river basins^25^. There is also no evidence of Mesozoic sediments of the Paraná basin (or Bauru basin) crossing the Canastra range^26^, the divide between the upper Paraná and São Francisco rivers. A compilation of available, mainly unpublished paleocurrent and provenance data^27–31^, for the late Cretaceous sedimentary units north and south of Azimuth 125° lineament shows a clear dispersion from this lineament, which behaved as a topographic high during sedimentation.

Relatively well-known major geological events, such as the opening of the South Atlantic Ocean (Jurassic to early Cretaceous) or the rise of the Andean chain (late Cretaceous to Cenozoic), can explain several aspects of the South American drainage evolution, particularly along the eastern, passive, rifted margin of the continent^23^ as well as on the opposite convergent Andean margin^32^. However, the origin of the present-day N-S compartmentalization of the drainage network requires further explanation with respect to the underlying combination of mechanisms involved.

### Heat source for intracontinental magmatic province formation

Heat source and faulting are important factors affecting the formation of intracontinental magmatic provinces, as here proposed to cause the formation of the long, South American transcontinental water divide. In this section, two proposed general alternative heat-source models are addressed: mantle plumes and tectonic reactivations.

Geologic, geomorphologic, and geochronologic evidence has been used to postulate that the alkaline rocks between Poços de Caldas (continental interior; Minas Gerais) and the Cabo Frio coast (Rio de Janeiro) have a WNW-ESE alignment and were emplaced during the displacement of the South American plate over the Trindade hot spot currently located at ~18°40′S in the Mid-Atlantic Ridge (mantle plume hypothesis)^33^. According to this view, during the Eocene, this supposedly existing hot spot probably moved to the eastern boundary (coast of Rio de Janeiro) of South America, causing important tectonic and magmatic events. This relative hot spot displacement has been considered to have caused the formation of the volcanic Vitória-Trindade chain, located off the eastern coast of Brazil, corresponding to the oceanic extension of the Azimuth 125° magmatic lineament. Furthermore, the genesis of the Poxoréu Igneous Province (Mato Grosso, western Brazil) has been also proposed to possibly be associated with a more intense lithospheric extension above the western margin of the postulated impact zone of the Trindade plume, permitting greater upwelling and melting farther to the west at ~84 Ma^34^. Therefore, according to this view, the Trindade plume was considered to possibly represent a super-plume with a diameter of ~1000 km, and the plume were thought to serve as heat sources for continental-interior igneous province formation. It is important to note, however, that the western end of the Vitoria-Trindade Chain is more than 280 km north of the southeastern end of the Azimuth 125° magmatic lineament. Moreover, the plume hypothesis has been criticized recently because geochemical data do not support that tholeiites from the Paraná Magmatic Province resulted from the Trindade plume^35^, and the oceanic crust was recently reactivated as well as subject to alternating compressive and extensional stresses associated with normal faulting and volcanism^36,37^.

Several supposedly existing “hotspot tracks”, such as the Vitória-Trindade chain, might reflect that the heat is derived from the accommodation of stresses in the lithosphere during rifting rather than continuous magmatic activity induced by mantle plumes beneath the moving lithospheric plates. Considering this view, regional thermal anomalies in the deep mantle, mapped using geoid and seismic tomography data, offer an alternative, non-plume-related heat source for the generation of intracontinental magmatic provinces^35^.

**Figure 3:**
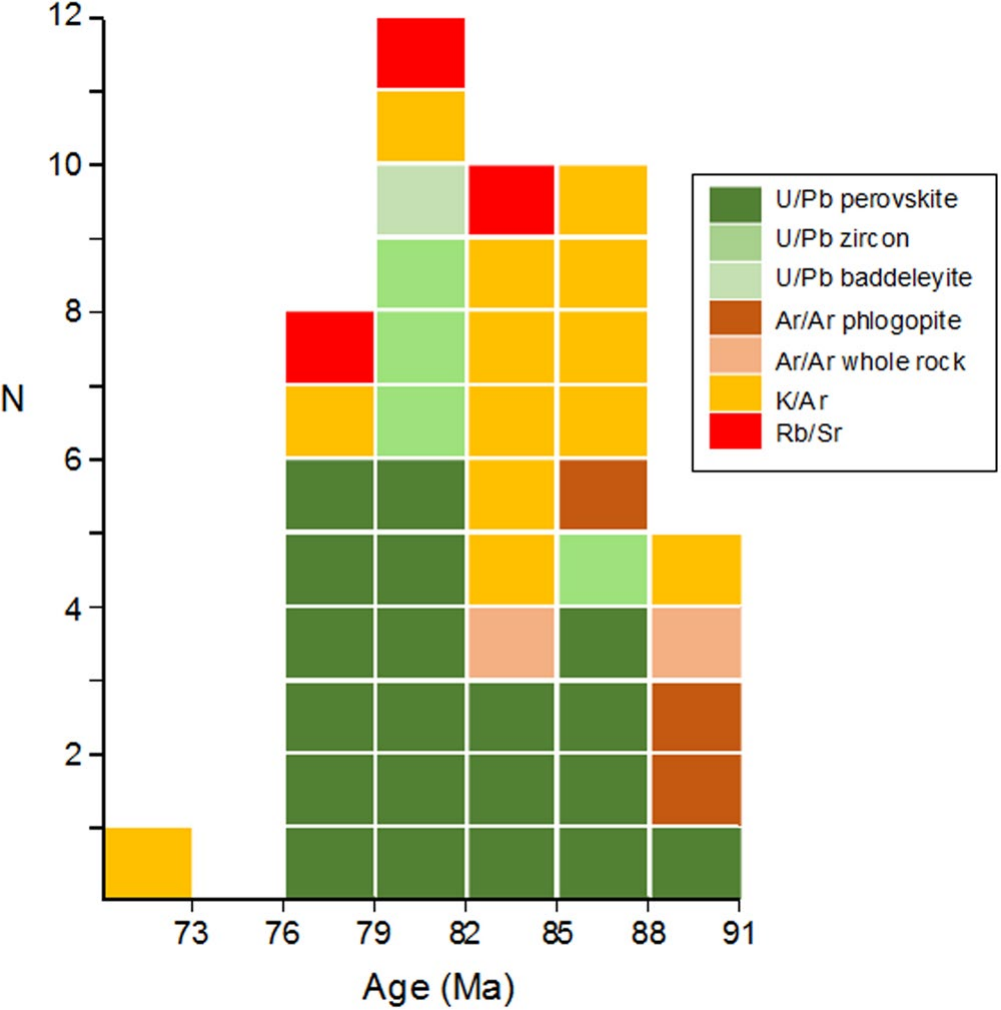
Histogram showing distribution (n) of alkaline rock ages along the Azimuth 125° magmatic lineament between Poxoréu (Mato Grosso) and Cabo Frio (Rio de Janeiro), Brazil.

The distribution of alkaline occurrences along NW-SE-trending crustal discontinuities extending over 800 km and the nature of the magmatism as described above clearly indicate that deep lithospheric faults significantly controlled the tectonics of the alkaline provinces in the Azimuth 125° lineament^38^. Alkaline bodies were emplaced between 91 and 72.4 Ma (97 and 71.1 Ma including uncertainty), with a higher concentration between 76 and 88 Ma (Fig. 3). The distribution of age-dates of the alkaline rocks along the Azimuth 125° does not show any eastward-decreasing trend. Instead, the available ages indicate a relatively long magmatic activity (~12 Ma) that weakens the hypothesis of the action of a mantle plume. In fact, available age data indicate the occurrence of different phases of alkaline magmatism from Late Cretaceous to Paleogene^38^. Thus, the supposed “impact of the Trindade starting mantle plume head”^34^ that developed at about 250 km west of the Poxoréu Igneous Province on intracontinental magmatic province formation has been perceived as “very improbable”^39^.

## Discussion

Is there a link between drainage compartmentalization and uplift controlled by intrusive magmatism? The magnetic signature of the Azimuth 125° lineament indicates a set of linear features with regional continuity in the subsurface, characterized by a higher magnetic susceptibility compared with surrounding host rocks^40^. The importance of this lineament as a system of deep crustal discontinuities serving as the main conduit for several alkaline intrusions along the azimuth axis has been confirmed recently^40^. The injection of dike-forming magma into the faults of the lineament occurred during two or three tectonic events: (i) between 950 and 520 Ma at two Brasiliano orogeny cycles, older (950–650 Ma) and younger (ca. 700–520 Ma); (ii) at approximately 180 Ma, during the fragmentation of Gondwana; and (iii) at circa 90 Ma^40^. A compilation of the available ages of intrusions along Azimuth 125° indicates periods of intrusions, and consequently, uplifts and large-scale drainage compartmentalization between 91–72.4 Ma (Fig. 3, Table 1).

**Table 1:** Available ages and nature of alkaline bodies along the Azimuth 125° magmatic lineament.

Alkaline rock occurrence	Rock	Age (Ma)	Error (Ma, 2σ)	Method	Dated material	Latitude	Longitude	Reference
Alpha 6	Kamafugite	79	3.1	Rb/Sr	phlogopite	18°31′10.24″S	46°48′16.67″W	^50^
Alpha 9	Kamafugite	80.1	4.6	U/Pb	perovskite	18°31′22.26″S	46°46′4.68″W	^50^
Amorinópolis	Kamafugite	72.4	1.3	K/Ar	whole rock	16°41′30.00″S	51°02′15.38″W	^51^
Araxá	Carbonatite, Glimmerite	77.4	1.0	K/Ar	phlogopite	19°40′15.82″S	46°56′51.67″W	^52^
Campos basin offshore	Gabbro	81	5	K/Ar	n.a.	22°19′41.25″S	40°08′42.60″W	^53^
Carmo do Parnaíba	Carbonatite, kimberlite, lamproite and kamafugitic rocks	83.6	1.4	K/Ar	phlogopite	19°01′54.10″S	46°17′41.21″W	^43^
Catalão I	Magnetitite	81	4	U/Pb	baddeleyite	18°08′4.84″S	47°48′37.06″W	^54^
Catalão II	Phlogopite-picrite	82	3	U/Pb	perovskite	18°02′51.40″S	47°52′34.03″W	^55^
Dourados 19	Kimberlite	76.9	4.1	U/Pb	perovskite	18°51′50.25″S	47°03′42.71″W	^50^
Esperança	Kimberlite	79.6	1.2	U/Pb	zircon	19°58′53.00″S	45°57′13.00″W	^56^
Indaiá	Kimberlite	80	5	U/Pb	perovskite	18°32′47.21″S	47°27′34.05″W	^55^
Iporá	Syenite, gabbro, piroxenite	82.9	3.1	K/Ar	biotite	16°26′S	51°02′″W	^52^
Japecanga 6	Kamafugite	84.5	0.9	Rb/Sr	phlogopite	18°24′48.26″S	47°21′39.85″W	^50^
Joana 2	Kamafugite	78.9	6.9	U/Pb	perovskite	20°03′18.33″S	46°53′57.70″W	^50^
Joana 5	Kamafugite	80.1	1.2	U/Pb	zircon	20°03′18.96″S	46°53′56.40″W	^56^
Joana 6	Kamafugite	79.2	1.2	U/Pb	zircon	20°03′12.25″S	46°54′6.65″W	^56^
Lemes	Kimberlite	84	2	U/Pb	perovskite	18°11′48.19″S	47°34′42.16″W	^55^
Limeira	Kimberlite	91	6	U/Pb	perovskite	18°30′49.18″S	47°31′27.57″W	^55^
Limpeza 18	Kimberlite	79.9	3.2	U/Pb	perovskite	18°28′51.42″S	46°47′53.20″W	^50^
Malaquias	Kamafugite and sub-volcanic phonolite	79	2	U/Pb	perovskite	18°26′13.77″S	46°18′09.73″W	^55^
Mata da Corda	Kamafugite	86.6	5.2	U/Pb	perovskite	18°55′S	46°13′W	^57^
Montes Claros de Goiás	Essexite, gabbro, syenite	88.7	5.7	K/Ar	biotite	16°04′S	51°23′W	^52^
Pântano	Kimberlite	83	2	U/Pb	perovskite	18°28′11.80″S	46°47′21.08″W	^55^
Paranaíba 14	Kamafugite	79.2	5.6	U/Pb	perovskite	18°51′18.95″S	46°14′25.87″W	^50^
Perdizes 2	Kimberlite	87.2	3.0	U/Pb	perovskite	18°33′2.25″S	47°28′1.77″W	^50^
Perdizes 3	Kimberlite	79	4	U/Pb	perovskite	18°34′15.62″S	47°27′27.78″W	^55^
Poço Verde	Kimberlite	86	1.2	U/Pb	zircon	18°23′22.77″S	47°11′41.83″W	^56^
Poços de Caldas	Tinguaite, phonolite, nepheline syenite and pyroclastic rocks	86.7	0.4	Ar/Ar	phlogopite	21°51′12.23″S	46°35′21.69″W	^58^
Ponta do Morro	Granite, quartz monzonite, syenite and nordmarkite	84	6	Rb/Sr	whole rock (syenite)	16°16′41.20″S	55°41′41.67″W	^59^
Ponte Nova	Gabbro, clinopyroxenite	87.6	1.3	K/Ar	biotite	22°47′21.24″S	45°45′10.19″W	^60^
Poxoréu	Basalt, syenite, monzonite, granite	83.9	0.4	Ar/Ar	whole rock	15°58′33.25″S	53°58′14.12″W	^34^
Presidente Olegário	Kamafugite	79	1	U/Pb	perovskite	18°24′15.74″S	46°28′32.43″W	^55^
Presidente Olegário 3	Kamafugite	83.9	5.2	U/Pb	perovskite	18°25′51.96″S	46°25′35.49″W	^50^
Rio Preto 2	Kamafugite	88.0	4.4	U/Pb	perovskite	18°17′25.62″S	47°23′40.70″W	^50^
Rio Verde 3	Kamafugite	88.24	0.56	Ar/Ar	phlogopite	17°03′20″S	51°09′W	^50^
Salitre	Bebedourite, phonolite	82.5 86.3	5.6 5.7	K/Ar K/Ar	biotite biotite	19°02′S	46°46′W	^52^
Santa Fé	Essexite, missourite, lamprophire, malignite	86.7	1.8	K/Ar	biotite	15°43′S	51°08′W	^52^
Santo Antônio da Barra	Kamafugite and sub-volcanic phonolite	77.9	6.8	U/Pb	perovskite	17°35′S	50°40′W	^57^
Serra Negra	Peridotite	83.5	n.a.	K/Ar	biotite	18°55′S	46°50′W	^52^
Serra do Bueno	Katungite, mafurite and ugandite (kamafugitic rocks)	90	4	Ar/Ar	whole rock	19°43′49.60″S	46°00′01.79″W	^61^
Tapira	Phonolite, jacupiranguite, bebedourite	87.2	1.2	K/Ar	biotite	19°52′41.71″S	46°51′5.68″W	^52^
Três Ranchos	Kimberlite	87	3	U/Pb	perovskite	18°20′19.67″S	47°51′34.05″W	^55^
Três Ranchos 4	Kimberlite	81.6	2.7	Rb/Sr	phlogopite	18°18′50.66″S	47°49′15.95″W	^50^
Três Ranchos 27	Kimberlite	81.1	6.6	U/Pb	perovskite	18°26′26.21″S	47°49′27.83″W	^50^
Três Ranchos 78	Kimberlite	88.3	1.0	Ar/Ar	phlogopite	18°15′43.19″S	47°40′40.80″W	^50^

Low temperature thermochronology, including apatite fission track analysis (AFT) and a minor set of apatite U-Th/He dating (AHe), indicate that the onshore coastal region of SE Brazil experienced cooling, uplift and exhumation between 100 and 70 Ma^41^. Up to 3 km of denudation was inferred^42^, but this is significantly attenuated to the continental interior. Some alkaline rocks along the Azimuth 125° have deep sources (up to 100 and 150 km for kamafugites and kimberlites, respectively)^43^. The 3D inversion of magnetic data demonstrated that alkaline intrusions along Azimuth 125° are shallow^44^. A large number of occurrences have associated hypabyssal and/or volcanic (lavas) equivalents, or even rocks subject to phreatomagmatic interactions, indicating shallow or near surface emplacement and a very low, long-term denudation rate since the Late Cretaceous.

Emplacement of intrusive bodies causes surface uplift, as observed in other regions of the world as forced folds with amplitudes related to intrusion thickness and length^45^. Some intrusions (Araxá, Catalão 1, Poços de Caldas, Serra Negra, Tapira) (Table 1) dragged the surrounding rocks, causing uplift. A conspicuous feature in the Araxá (see map in^46^) and Serra Negra intrusions^47^ is the presence of a ring of Precambrian schists and quartzites that surround the alkaline rock body. In Poços de Caldas^48^ part of the roof (Early Cretaceous eolian sandstone) is preserved. Outcropping alkaline bodies show a maximum depth/major axis of 4.5/4.5 km for Araxá, 17/9 km for Tapira, 12–15/10 km for Serra Negra-Salitre and 5/5 km for Catalão 1, and alkaline bodies without surface manifestation show a minimum depth/major axis of 0.3–2/6 km for Pratinha and <2/14 km for Registro do Araguaia^44^. At the southwestern border of the Parecis Basin, along Azimuth 125°, a set of currently shallow intrusive bodies were identified from magnetic anomalies, having maximum length and thickness of approximately 11 and 3.6 km, respectively^49^. These dimensions suggest that, at the time of its placement, the surface of the terrain experienced a probable uplift of 0.1 to 1 km^45^. Although the minimum value was 100 m, this uplift is considered to be appreciable and is likely to have caused a change in the drainage network.

Here we show that uplift associated with late Cretaceous (91–72.4 Ma) intrusive magmatism explains the origin and maintenance of the present-day 2,300 km long, NW-SE-oriented water divide between the Amazonian, Paraná-Paraguay, and São Francisco river basins. Independent of the underlying mechanism (mantle plumes or tectonic reactivations), high cratonic topography arose from intracontinental magmatic activities in South America^19^. This scenario, along with several other completely different mechanisms (such as the Andean orogeny, large-scale foreland basins subsidence, marine incursions, the rise and disappearance of mega-wetlands, and erosive and tectonic headwater captures) illustrate the splendorous South American geodiversity acting on river basins throughout history.

## Methods

Geological data were collected from the literature. Intrusive alkaline complexes (carbonatite, kimberlite, and syenite) were also mapped using CPRM data (Brazilian Geological Survey) available on http://geosgb.cprm.gov.br/. Mapping were performed using QGIS v2.18 (http://www.qgis.org). The ages of the alkaline rocks were obtained from different sources (listed in Table 1), and mainly comprise U-Pb, Ar-Ar and few K-Ar and Rb/Sr data.

## References

[1] Potter PE (2003). Potter 1997 The Mesozoic and Cenozoic paleodrainage of South America a natural history. J. South Am. Earth Sci..

[2] Cox KG (1989). The role of mantle plumes in the development of continental drainage patterns. Nature.

[3] Colli L, Ghelichkhan S, Bunge H-P (2016). On the ratio of dynamic topography and gravity anomalies in a dynamic Earth. Geophys. Res. Lett..

[4] Rodríguez Tribaldos V, White NJ, Roberts GG, Hoggard MJ (2017). Spatial and temporal uplift history of South America from calibrated drainage analysis. Geochemistry, Geophys. Geosystems.

[5] Lovejoy NR, Albert JS, Crampton WGR (2006). Miocene marine incursions and marine/freshwater transitions: Evidence from Neotropical fishes. J. South Am. Earth Sci..

[6] Hovikoski Jussi, Wesselingh Frank P., Räsänen Matti, Gingras Murray, Vonhof Hubert B. (2011). Marine influence in Amazonia: Evidence from the Geological Record. Amazonia: Landscape and Species Evolution.

[7] Hoorn Carina, Wesselingh Frank P., Hovikoski Jussi, Guerrero Javier (2011). The Development of the Amazonian Mega-Wetland (Miocene; Brazil, Colombia, Peru, Bolivia). Amazonia: Landscape and Species Evolution.

[8] Lundberg, J. G. *et al*. The stage for Neotropical Fish Diversification: A history of tropical South American Rivers. In *Phylogeny and Classification of Neotropical* Fishes (eds Malabarba, L. R., Reis, R. E., Vari, R. P., de Lucena, Z. M. S. & Lucena, C. A. S.) 603 (Edipucrs, 1998).

[9] Hoorn Carina, Roddaz Martin, Dino Rodolfo, Soares Emilio, Uba Cornelius, Ochoa-Lozano Diana, Mapes Russell (2011). The Amazonian Craton and its Influence on Past Fluvial Systems (Mesozoic-Cenozoic, Amazonia). Amazonia: Landscape and Species Evolution.

[10] Wilkinson M. Justin, Marshall Larry G., Lundberg John G., Kreslavsky Mikhail H. (2011). Megafan Environments in Northern South America and their Impact on Amazon Neogene Aquatic Ecosystems. Amazonia: Landscape and Species Evolution.

[11] Mora Andres, Baby Patrice, Roddaz Martin, Parra Mauricio, Brusset Stéphane, Hermoza Wilber, Espurt Nicolas (2011). Tectonic History of the Andes and Sub-Andean Zones: Implications for the Development of the Amazon Drainage Basin. Amazonia: Landscape and Species Evolution.

[12] Ribeiro AC (2013). Distributions and phylogeographic data of rheophilic freshwater fishes provide evidences on the geographic extension of a central-Brazilian amazonian palaeoplateau in the area of the present day Pantanal Wetland. Neotrop. Ichthyol..

[13] Assine Mario L., Merino Eder R., Pupim Fabiano N., Warren Lucas V., Guerreiro Renato L., McGlue Michael M. (2015). Geology and Geomorphology of the Pantanal Basin. The Handbook of Environmental Chemistry.

[14] Assine Mario Luis, Merino Eder Renato, Pupim Fabiano do Nascimento, Macedo Hudson de Azevedo, Santos Mauricio Guerreiro Martinho dos (2015). The Quaternary alluvial systems tract of the Pantanal Basin, Brazil. Brazilian Journal of Geology.

[15] Sacek V (2014). Drainage reversal of the Amazon River due to the coupling of surface and lithospheric processes. Earth Planet. Sci. Lett..

[16] Cordani, U. G. & Sato, K. Crustal evolution of the South American Platform, based on Nd isotopic systematics on granitoid rocks. *Episodes* **22**, 167–173 (1999).

[17] AB'SÁBER AZIZ NACIB (2000). SUMMIT SURFACES IN BRAZIL. Revista Brasileira de Geociências.

[18] Zalán, P. V. & Oliveira, J. A. B. Origem e evolução do Sistema de Riftes Cenozóicos do Sudeste do Brasil. *Bol. Geociencias da Petrobras* **13**, 269–300 (2005).

[19] Hu J (2018). Modification of the Western Gondwana craton by plume-lithosphere interaction. Nat. Geosci..

[20] Bardet M (1977). Geólogie du diamante. Troisième partie: Gisementes de diamants d’Asie, d’Amérique, d’Europeet d’Australasie. Mémoires du Bur. Res. Geol. Min..

[21] Gonzaga, G. M. & Tompkins, L. A. Geologia do diamante. In *Principais depósitos minerais do Brasil, Parte A, vol. IV* (eds. Schobbenhaus, C., Queiroz, E. T. & Coelho, C. E. S.) 53–116 (DNPM-CPRM, 1991).

[22] Pereira RM (2008). Dispersão da picroilmenita: estudo de caso aplicado ao kimberlito Cancã, Ilicínea, Minas Gerais. Geociências.

[23] Ribeiro AC (2006). Tectonic history and the biogeography of the freshwater fishes from the coastal drainages of eastern Brazil: An example of faunal evolution associated with a divergent continental margin. Neotrop. Ichthyol..

[24] Ab’Sáber, A. N. O relevo brasileiro e seus problemas. In *Brasil: a terra e o homem* (ed. Azevedo, A. D.) 135–250 (Companhia Editora Nacional, 1964).

[25] Pedreira, A. J. & Bahia, R. B. C. Estratigrafia e evolução da bacia dos Parecis, região amazônica, Brasil: integração e síntese de dados dos projetos Alto Guaporé, Serra Azul, Serra do Roncador, Centro-Oeste de Mato Grosso e Sudeste de Rondônia. CPRM-Serviço Geológico do Brasil, **39** (2004).

[26] Petri, S. & Fulfaro, V. J. *Geologia do Brasil: Fanerozóico*. (EDUSP, 1983).

[27] Souza Jr., J. J. O. O Grupo Bauru na porção setentrional da Bacia Sedimentar do Paraná. In *Congresso Brasileiro de Geologia*, 32 944–953 (Sociedade Brasileira de Geologia, 1984).

[28] Coimbra, A. M. Concurso para obtenção de título de Livre-Docente junto ao Departamento de Paleontologia e Estratigrafia do lnstituto de Geociências da Universidade de São Paulo na Área do Conhecimento Sedimentologia: Sistematização Crítica da Obra. (Universidade de São Paulo, 1991).

[29] Weska RK (2006). Uma síntese do cretáceo superior Mato-Grossense. Geociencias.

[30] Fernandes, L. A. Estratigrafia e evolução geológica da parte oriental da Bacia Bauru (Ks, Brasil). (Universidade de São Paulo, 1998).

[31] Batezelli, A. Análise da sedimentação cretácea no triângulo mineiro e sua correlação com áreas adjacentes. (Universidade Estadual Paulista (UNESP), 2003).

[32] Lima, F. C. T. & Ribeiro, A. C. Continental-scale tectonic controls of biogeography and ecology. in *Historical Biogeography of Neotropical* Freshwater Fishes (eds Albert, J. & Reis, R. E.) 145–164 (University of California Press, 2011).

[33] Thomaz Filho A, Rodrigues AL (1999). O alinhamento de rochas alcalinas Poços de Caldas-Cabo Frio (RJ) e sua continuidade na cadeia Vitória-Trindade. Rev. Bras. Geociências.

[34] Gibson SA, Thompson RN, Weska RK, Dickin AP, Leonardos OH (1997). Late Cretaceous rift-related upwelling and melting of the Trindade starting mantle plume head beneath western Brazil. Contrib. to Mineral. Petrol..

[35] Ernesto M (2002). Paraná Magmatic Province-Tristan da Cunha plume system: Fixed versus mobile plume, petrogenetic considerations and alternative heat sources. J. Volcanol. Geotherm. Res..

[36] Ferrari LA, Riccomini C (1999). Campo de esforços Plio-Pleistocênico na Ilha da Trindade (Oceano Atlântico Sul, Brasil) e sua relação com a tectônica regional. Rev. Bras. Geociencias.

[37] Da Costa Alves E, Maia M, Sichel SE, De Campos CMP (2006). Zona de fratura de vitória-trindade no oceano atlântico sudeste e suas implicações tectônicas. Rev. Bras. Geofis..

[38] Riccomini, C., Velázquez, V. F. & Gomes, C. B. Tectonic controls of the Mesozoic and Cenozoic alkaline magmatism in the central-southeastern Brazilian Platform. In *Mesozoic and Cenozoic Alkaline* Magmatism *in the Brazilian Platform* (eds Comin Chiaramonti, P. & Gomes, C. B.) 31–56 (EDUSP, 2005).

[39] Comin-Chiaramonti P (2002). Geochemistry and geodynamic implications of the Anitápolis and Lages Alkaline-Carbonatite complexes, Santa Catarina State, Brazil. Rev. Bras. Geociências.

[40] Moraes Rocha LG, de, Pires ACB, Carmelo AC, Araújo Filho JO (2014). de. Geophysical characterization of the Azimuth 125° lineament with aeromagnetic data: Contributions to the geology of central Brazil. Precambrian Res..

[41] Cogné Nathan, Gallagher Kerry, Cobbold Peter R., Riccomini Claudio, Gautheron Cecile (2012). Post-breakup tectonics in southeast Brazil from thermochronological data and combined inverse-forward thermal history modeling. Journal of Geophysical Research: Solid Earth.

[42] Hiruma ST (2010). Denudation history of the Bocaina Plateau, Serra do Mar, southeastern Brazil: Relationships to Gondwana breakup and passive margin development. Gondwana Res..

[43] Gibson SA, Thompson RN, Leonardos OH, Dickin AP, Mitchell JG (1995). The late cretaceous impact of the trindade mantle plume: Evidence from large-volume, mafic, potassic magmatism in SE Brazil. J. Petrol..

[44] Marangoni YR, Mantovani MSM (2013). Geophysical signatures of the alkaline intrusions bordering the Paraná Basin. J. South Am. Earth Sci..

[45] Magee C (2017). Structure and dynamics of surface uplift induced by incremental sill emplacement. Geology.

[46] Traversa G (2001). Petrography and mineral chemistry of carbonatites and mica-rich rocks from the Araxá complex (Alto Paranaíba Province, Brazil). An. Acad. Bras. Cienc..

[47] Grasso, C. B. Petrologia do Complexo Alcalino- Carbonatítico de Serra Negra, MG. (Universidade de Brasília, 2010).

[48] Ulbrich, H. H., Vlach, S. R. F., Demaiffe, D. & Ulbrich, M. N. C. Structure and origin of the Poços de Caldas alkaline massif, SE Brazil. In *Mesozoic to Cenozoic Alkaline* Magmatism *In the Brazilian Platform* (eds Comin-Chiaramonti, P. & Gomes, C. de B.) 367–418 (FAPESP, 2005).

[49] Ribeiro VB, Louro VHA, Mantovani MSM (2013). 3D Inversion of magnetic data of grouped anomalies - Study applied to São José intrusions in Mato Grosso, Brazil. J. Appl. Geophys..

[50] Felgate, R. M. The Petrogenesis of Brazilian kimberlites and kamafugites intruded along the 125° lineament - Improved geochemical and geochronological constraints on magmatism in Rondonia and the Alto Paranaiba igneous province. (The University of Melbourne, 2014).

[51] Danni JC, Gasper JC (1994). Química do Katungito de Amorinópolis - Goiás: Contribuição ao estudo do magmatismo kamafugítico. Geochim. Bras..

[52] Sonoki IK, Garda GM (1988). Idades K-Ar de rochas alcalinas do Brasil meridional e Paraguai oriental: compilação e adaptação às novas constantes de decaimento. Bol. IG-USP. Série Científica.

[53] ALMEIDA FERNANDO FLÁVIO MARQUES DE, CARNEIRO CELSO DAL RÉ , MIZUSAKI ANA MARIA PIMENTEL  (1996). CORRELAÇÃO DO MAGMATISMO DAS BACIAS DA MARGEM CONTINENTAL BRASILEIRA COM O DAS ÁREAS EMERSAS ADJACENTES. Revista Brasileira de Geociências.

[54] Guarino Vincenza, Wu Fu-Yuan, Melluso Leone, de Barros Gomes Celso, Tassinari Colombo Celso Gaeta, Ruberti Excelso, Brilli Mauro (2016). U–Pb ages, geochemistry, C–O–Nd–Sr–Hf isotopes and petrogenesis of the Catalão II carbonatitic complex (Alto Paranaíba Igneous Province, Brazil): implications for regional-scale heterogeneities in the Brazilian carbonatite associations. International Journal of Earth Sciences.

[55] Guarino Vincenza, Wu Fu-Yuan, Lustrino Michele, Melluso Leone, Brotzu Pietro, Gomes Celso de Barros, Ruberti Excelso, Tassinari Colombo Celso Gaeta, Svisero Darcy Pedro (2013). U–Pb ages, Sr–Nd- isotope geochemistry, and petrogenesis of kimberlites, kamafugites and phlogopite-picrites of the Alto Paranaíba Igneous Province, Brazil. Chemical Geology.

[56] Davis GL (1977). The ages and uranium contents of zircons from kimberlites and associated rocks. Carnegie Inst. Washingt. Yearb..

[57] Sgarbi Patrícia B.A, Heaman Larry M, Gaspar José Carlos (2004). U–Pb perovskite ages for brazilian kamafugitic rocks: further support for a temporal link to a mantle plume hotspot track. Journal of South American Earth Sciences.

[58] Vlach Silvio Roberto Farias, Ulbrich Horstpeter Herberto Gustavo Jose, Ulbrich Mabel Norma Costas, Vasconcelos Paulo Marcos (2018). Melanite-bearing nepheline syenite fragments and 40Ar/39Ar age of phlogopite megacrysts in conduit breccia from the Poços de Caldas Alkaline Massif (MG/SP), and implications. Brazilian Journal of Geology.

[59] Del’Arco, J. O. *et al*. *Projeto Radambrasil: Folha SE21 Corumbá e parte da folha SE 20, geologia, geomorfologia, pedologia, vegetacão e uso potencial da terra* (1982).

[60] Azzone RG, Ruberti E, Rojas GEE, De Barros Gomes C (2009). Geologia e geocronologia do maciço alcalino máfico- ultramáfico Ponte Nova (SP-MG). Geol. USP - Ser. Cient..

[61] Gibson S. A., Thompson R. N., Leonardos O. H., Turner S. E., Mitchell J. G., Dickin A. P. (1994). The Serra do Bueno potassic diatreme: a possible hypabyssal equivalent of the ultramafic alkaline volcanics in the Late Cretaceous Alto Paranaίba Igneous Province, SE Brazil. Mineralogical Magazine.

